# Regional and Longitudinal Dynamics of Cyanobacterial Blooms/Cyanobiome and Cyanotoxin Production in the Great Lakes Area

**DOI:** 10.3390/toxins16110471

**Published:** 2024-11-01

**Authors:** Faizan Saleem, Jennifer L. Jiang, Enze Li, Kevin Tran, Adam Boere, Mahbuba Rahman, Athanasios Paschos, Judy A. Westrick, Arthur Zastepa, Thomas A. Edge, Herb E. Schellhorn

**Affiliations:** 1 Department of Biology, McMaster University, Hamilton, ON L8S 4L8, Canada; jiangl55@mcmaster.ca (J.L.J.); lie40@mcmaster.ca (E.L.); trank38@mcmaster.ca (K.T.); boerea1@mcmaster.ca (A.B.); rahmm16@mcmaster.ca (M.R.); ethan.paschos@mohawkcollege.ca (A.P.); edget2@mcmaster.ca (T.A.E.); 2 Lumigen Instrument Center, Wayne State University, 5101 Cass Ave., Detroit, MI 48202, USA; judy.westrick@wayne.edu; 3 Environment and Climate Change Canada, Canada Centre for Inland Waters, 867 Lakeshore Road, Burlington, ON L7S 1A1, Canada; arthur.zastepa@ec.gc.ca

**Keywords:** the Great Lakes, DNA sequencing, harmful algal blooms, cyanobacteria, molecular methods, metabarcoding

## Abstract

Cyanobacteria (blue-green algae) are a diverse group of prokaryotic microorganisms that impact global biogeochemical cycles. Under eutrophic conditions, cyanobacterial species can produce cyanotoxins, resulting in harmful algal blooms (cHABs) that degrade water quality and result in economic and recreational losses. The Laurentian Great Lakes, a key global freshwater source, are increasingly affected by these blooms. To understand the underlying mechanisms in cHAB formation, we investigated microcystin levels, cyanotoxin genes/transcripts, and taxonomic/microcystin metabarcoding across three sampling locations in the Canadian Great Lakes region, including Hamilton Harbour, Bay of Quinte, and Three Mile Lake (Muskoka), to observe the regional and longitudinal cyanobacterial dynamics. The results revealed a positive correlation between microcystin levels, the occurrence of cyanobacterial taxonomic/cyanotoxin molecular markers, and the relative widespread abundance of specific dominant cyanobacterial taxa, including *Planktothrix*, *Microcystis*, and *Dolichospermum*. The *Cyanobium* genus was not observed in Hamilton Harbor samples during late summer (August to September), while it was consistently observed in the Three Mile Lake and Bay of Quinte samples. Notably, *Dolichospermum* and saxitoxin genes were predominantly higher in Three Mile Lake (an inland lake), suggesting site-specific characteristics influencing saxitoxin production. Additionally, among the potential microcystin producers, in addition to *Microcystis*, Hamilton Harbour and Bay of Quinte samples showed consistent presence of less dominant microcystin-producing taxa, including *Phormidium* and *Dolichospermum*. This study highlights the complexity of cHAB formation and the variability in cyanotoxin production in specific environments. The findings highlight regional and site-specific factors that can influence cyanobacterial taxonomic and molecular profiles, necessitating the integration of advanced molecular technologies for effective monitoring and targeted management strategies.

## 1. Introduction

Cyanobacteria, or blue-green algae, are a phylogenetically diverse group of prokaryotic microorganisms capable of oxygenic photosynthesis [1,2]. The photosynthetic capability of cyanobacteria influences biogeochemical cycles by modulating environmental oxygen levels and contributing to the nitrogen cycle through nitrogen fixation [3]. Despite the ecological importance of cyanobacteria, under eutrophic conditions [4], specific cyanobacterial species can synthesize toxic secondary metabolites, i.e., cyanotoxins, which result in deterioration of water quality because of cyanobacterial harmful algal bloom (cHAB) formation and consequently lead to recreational and economic losses [5]. The economic losses due to cHAB formation include but are not limited to lost recreational revenue, reduced property values, and elevated costs for drinking water treatment [6]. Although extensive literature is available on cyanobacteria, the underlying mechanisms involved in the onset, dominance, and transformation into cHABs can be complex [7]. Among environments prone to cHAB formation, the conservation of global water reservoirs, including the Great Lakes, is paramount because of their role in providing drinking water, supporting diverse ecosystems, and sustaining economic activities like fishing and tourism.

The Laurentian Great Lakes of North America collectively hold around 21% of the world’s freshwater and 84% of North America’s surface water supply [8]. The Great Lakes comprise five lakes spanning over 200,000 km^2^ and cross the borders of the United States and Canada [9]. Nearly 30% of the economies in both nations are linked to the Great Lakes through fisheries, industry, tourism, and recreational activities [9]. Among the environmental factors associated with the deterioration of the Great Lakes, cHABs are of priority. Because of its relatively shallow depth and moderate climate, Lake Erie is particularly prone to significant seasonal cHABs [10], and despite extensive restoration efforts in the late 1900s, there has been a resurgence of cyanobacterial harmful algal blooms (cHABs) in several densely populated regions of the Great Lakes [10]. While Lake Erie is known for having the most extensive bloom events among the Great Lakes, cHABs and associated cyanotoxins are now found throughout all of the Great Lakes [11,12,13].

Cyanobacteria and cyanotoxin indicators, including phycocyanin, total cyanobacteria cell counts, and cyanotoxin levels, are often analyzed to assess the cyanotoxicity of recreational waters [14]. However, cHAB testing programs usually incorporate only single or limited numbers of cyanobacterial markers, which may not provide sufficient information regarding regional or environmentally specific changes and can undermine the detection of less dominant/frequent cHAB-associated factors. Conventional strategies for cyanobacterial and cyanotoxin monitoring in the Great Lakes include microscopy [15], enzyme-linked immunosorbent assays (ELISAs) [16], and chromatography coupled with mass spectrometry [17]. However, information obtained from conventional methods may be limited and may lack specificity/sensitivity for detecting less dominant cyanobacterial indicators. Molecular technologies, including next-generation DNA sequencing and diagnostic DNA amplification (PCR/qPCR), can potentially augment existing cyanobacterial monitoring strategies by providing more robust and comprehensive taxonomic/functional profiles [18]. Studies on cHABs are mostly information-limited because of the reliance on conventional technologies, lack of comparison between recreational waters with different ecological/environmental settings, and limited sampling plans. This study assessed regional and environmentally specific cyanobacterial diversity and the underlying taxonomic dynamics associated with changes in conventional cyanobacterial/cyanotoxin indicators. Additionally, we assessed three recreational water bodies with different ecological/urban settings throughout the summer using emerging molecular technologies, including DNA amplification, ELISA, and DNA sequencing, to obtain a comprehensive profile of taxonomic and functional changes associated with cHAB formation. The major questions explored in this study are as follows. (1) Is the cyanobacterial diversity similar between different Great Lakes regions? (2) Are there any cyanobacterial changes that are site-/regionally specific? (3) Are microcystin levels correlated with the presence of other less dominant cyanotoxins? (4) Is there any relationship between cyanobacterial taxa occurrence and taxonomic/cyanotoxin molecular markers? (5) Which cyanobacterial taxa are potential toxin producers or are associated with cyanotoxicity?

## 2. Results

### 2.1. Quality Control Analysis

Quality control analysis was employed in each molecular method to isolate bias associated with experimental procedures or handling. For microcystin ELISA, the mean recovery of the seven LFBs was 117% ± 13%, indicating a recovery rate in an acceptable range. To confirm the minimum reporting limit (0.4 ng/mL), the upper prediction interval result (PIR) limit was 115%, and the lower PIR limit was 54%. For ELISA assays, a correlation coefficient (r^2^) of 0.99 was observed for the calibration curves. The microcystin standard had a coefficient of variation (CV) of less than 10%. For cyanotoxin qPCR assays, the correlation coefficient (r^2^) for each qPCR assay was around 0.99. The intercept values for the standard curves varied between 38.5 and 39.1, while the slope values ranged from −3.2 to −3.4. The calculated efficiencies of the standard curves spanned from 94% to 101%.

For taxonomic and microcystin metabarcoding, 95 samples were amplified and sequenced for *16S* taxonomic analysis, whereas 47 samples corresponding to positive *mcyE* gene amplification were analyzed to identify potential microcystin-producing cyanobacterial taxa (microcystin metabarcoding). Deep next-generation sequencing yielded an average of 329,062 ± 96,959 reads per *16S* rRNA library and 209,861 ± 59,343 reads per *mcyE* library. After quality filtration, 69% of the *16S* rRNA reads and only 17% of the *mcyE* reads remained.

### 2.2. Trends in Microcystin and Cyanotoxin Distributions

**Figure 1:**
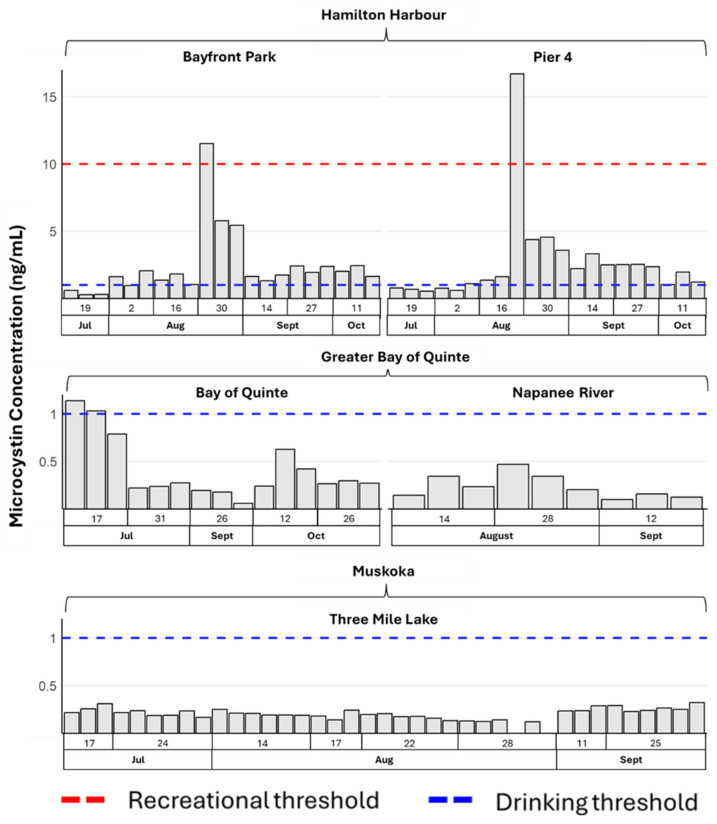
Microcystin levels (ng/mL) at all sampling locations throughout the sampling period. The red dotted line indicates the WHO recreational/contact guideline of 10.0 ng/mL, while the blue dotted line marks the WHO drinking water guideline of 1.0 ng/mL.

All the samples were first tested for microcystin levels, and 50 positive samples were analyzed for cyanobacteria and cyanotoxin gene copies to understand the dynamic trends in cyanobacteria/cyanotoxin throughout the summer. All the samples except for two from Three Mile Lake (Muskoka) were positive for microcystin (Figure 1). Compared with other sampling locations, microcystin was detected more frequently in Hamilton Harbour sites, including Bayfront Park and Pier 4. Specifically, 81% of the Bayfront Park samples, 76% of the Pier 4 samples, and 13% of the Bay of Quinte samples exceeded the WHO drinking water quality threshold (1 ng/mL) (WHO, 2022), while all samples from Three Mile Lake were below drinking water quality thresholds.

For cyanotoxin (microcystin, cylindrospermopsin, and saxitoxin) qPCR analysis, 50 samples in total were analyzed for all the sampling locations (Supplementary Figure S1). All the sampling sites were negative for the cylindrospermopsin gene (*cyrA*), while all the tested samples from Hamilton Harbour and Bay of Quinte were positive for microcystin gene (*mcyE*) copies. We did not observe any significant (*p* = 0.21) difference in microcystin gene copies between the Hamilton Harbor and Bay of Quinte samples (Supplementary Figure S1). However, the samples from Three Mile Lake (Muskoka) had significantly (*p* < 0.01) lower microcystin gene copies compared with both the Hamilton Harbour and Bay of Quinte sampling sites (Supplementary Figure S1). Similar to the DNA information, microcystin gene transcripts, i.e., RNA, showed no significant difference (*p* = 0.09) between the Bay of Quinte and Hamilton Harbour sampling sites, while microcystin transcripts were significantly (*p* < 0.01) lower in the Three Mile Lake (Muskoka) sampling sites (Supplementary Figure S2). Interestingly, the saxitoxin gene was only detected in the Three Mile Lake (Muskoka) samples but not in the Bay of Quinte and Hamilton Harbour samples (Supplementary Figure S3). Additionally, cyanobacterial *16S* gene copies were consistently present throughout the sampling season (Supplementary Figure S4), and no significant difference (*p* = 0.31) was observed between the sampling sites.

### 2.3. Identification of Microcystin Congeners and Cyanotoxins

Mass spectrometric analysis was used to identify the types of microcystin congeners and other cyanotoxins occurring at the tested sites (Table 1). The Hamilton Harbour sites showed the most diverse microcystin/cyanotoxin profile, with microcystin-RR (95%) as the most frequently detected microcystin congener, followed by microcystin-LR (89%), microcystin-YR (79%), and microcystin-LA (5%). Anabaenopeptin congeners, anabaenopeptin A and B and oscillamide Y, and anatoxin were also detected in the Hamilton Harbour samples. Bay of Quinte only showed the presence of microcystin-RR (30%), microcystin-LR (30%), and microcystin-LA (10%), while none of the tested cyanotoxins were detected in the Three Mile Lake samples by mass spectrometry.

### 2.4. Relationship Between Microcystin and Cyanobacteria/Cyanotoxin Gene Markers

Correlation analysis was performed on data compiled for all the sampling sites to understand the relationship between different cyanobacterial indicators (Table 2). Microcystin concentration positively correlated with *mcyE* gene copies (r_p_ = 0.71, *p* = 7.9 × 10^−7^), cyanobacteria *16S* gene copies (r_p_ = 0.5, *p* = 1.4 × 10^−3^), and *mcyE* transcripts (r_p_ = 0.5, *p* = 4.4 × 10^−3^). However, microcystin concentrations did not correlate with saxitoxin (*sxtA*) gene copies (r_p_ = 0.2, *p* = 0.3). Additionally, microcystin (*mcyE*) transcripts were significantly and positively correlated with microcystin gene copies (r_p_ = 0.6, *p* = 5.8 × 10^−4^) but showed no significant correlation with cyanobacteria *16S* gene copies (r_p_ = 0.3, *p* = 0.08) or saxitoxin gene (*sxtA*) copies (r_p_ = 0.05, *p* = 0.72).

### 2.5. Microbiome and Cyanobiome Diversity Analysis

**Figure 2:**
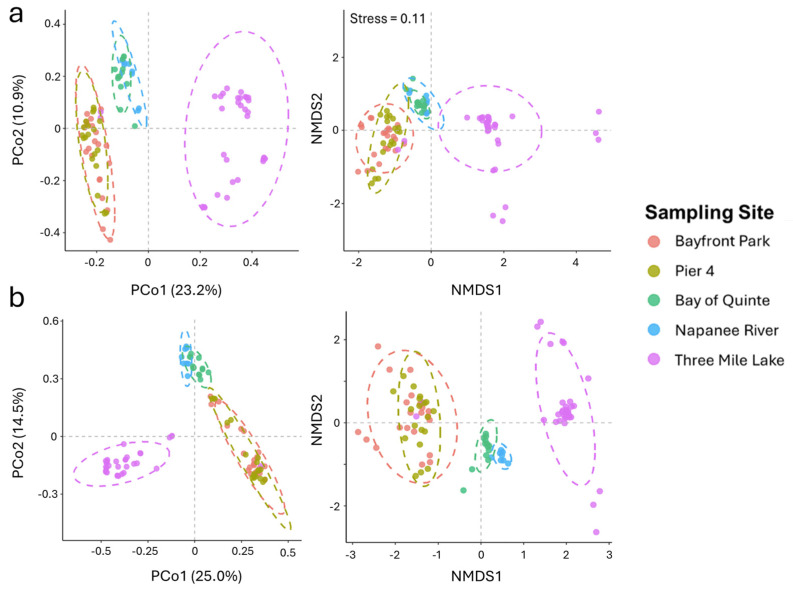
Beta diversity analysis of samples from all sampling locations. (**a**) Beta diversity based on the whole microbiome and (**b**) beta diversity based on cyanobiome.

Alpha (within the samples) and beta (among the samples) diversity analysis was assessed using the whole microbiome (Supplementary Table S1) and cyanobiome (Supplementary Table S2) to characterize the overall microbial/cyanobacterial similarity/dissimilarity associated with the tested sites/locations. Alpha diversity measurements were consistent among the sampling sites on the whole microbiome level (Supplementary Table S1). However, when resolved using just cyanobiome amplicons, the Hamilton Harbour sites showed a comparatively lower number of identified taxa and alpha diversity than the other sampling sites/locations (Supplementary Table S2). Core microbiome analysis also showed that 6% of the identified amplicon sequence variants (ASVs) were shared between the sampling sites (Supplementary Figure S5), with Three Mile Lake (Muskoka) showing the highest number of unique ASVs (35%), followed by Hamilton Harbour (30%) and Bay of Quinte (18%). Additionally, the water samples from Three Mile Lake (Muskoka) were differentiated into separate clusters and were more randomly distributed (Figure 2), while sampling sites from close geographical locations clustered in close proximity to each other.

### 2.6. Whole Microbiome and Cyanobiome Dynamics

Major microbial taxonomic groups were identified for all sampling sites to understand the changes in the whole microbiome and cyanobiome specific to each site and throughout the summer. Proteobacteria (25–26%), Actinobacteria (15–24%), and Cyanobacteria (10–18%) were the most dominant bacterial phyla for all the sampling sites and throughout the summer (Supplementary Figure S6). The cyanobacterial phylum was resolved to the genus level to understand the cyanobiome-specific changes in water samples (Supplementary Figure S7). *Planktothrix* was consistently the most dominant cyanobacterial genus in all sampling locations throughout the summer, with an overall relative abundance of ~33%, while other cyanobacterial genera demonstrated temporal and longitudinal distribution. Specifically, *Vulcanococcus* was the predominant genus in Hamilton Harbour for the samples from July before being overtaken by *Lyngbya* in August. *Microcystis* relative abundance remained consistent for Hamilton Harbour and Bay of Quinte sites throughout the summer. Compared with the Hamilton Harbour sites, *Cyanobium* relative abundance was higher and consistently distributed throughout the summer for the Bay of Quinte and Three Mile Lake (Muskoka) sites. *Dolichospermum* was the most dominant species at Three Mile Lake (Muskoka) throughout the summer compared with the other tested sites.

**Figure 3:**
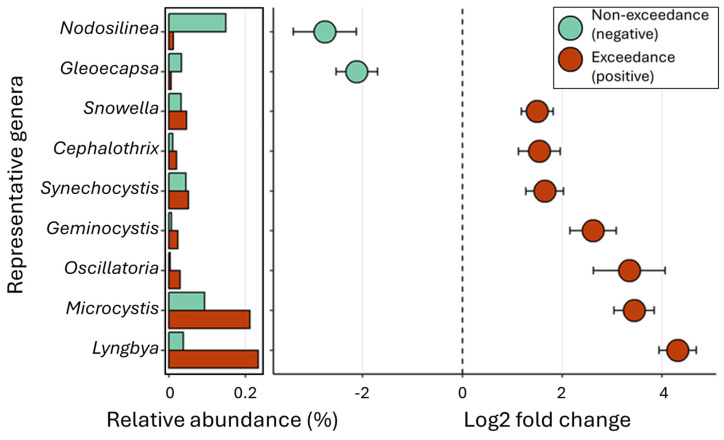
Differential abundance of cyanobacterial genera in microcystin exceedance and non-exceedance samples. Samples were categorized as exceedance and non-exceedance based on the WHO drinking water quality threshold (1 ng/mL) and with a *p*-value cut-off of <0.05.

Changes in the abundance of cyanobacterial genera were assessed to understand the role of identified cyanobacteria in microcystin production. Blooms dominated by *Microcystis* and *Lyngbya* showed the highest microcystin concentration, followed by *Oscillatoria*, *Geminocystis*, *Snowella*, *Cephalothrix*, and *Synechocystis* (Figure 3). To understand the relationship between the relative abundance of cyanobacterial genera and cyanobacterial-specific molecular markers (Supplementary Figure S8), we performed a correlation analysis. Twelve of the identified cyanobacterial genera positively correlated with microcystin levels, *mcyE* gene copies, and *mcyE* transcripts. *Dolichospermum*, *Pseudanabaena*, *Nodosilinea*, and *Cyanobium* (four of the six cyanobacterial genera in Three Mile Lake) exhibited a negative correlation with microcystin molecular markers but were positively correlated with *sxtA* gene copies.

### 2.7. Characterization of Microcystin-Producing Cyanobiome

**Figure 4:**
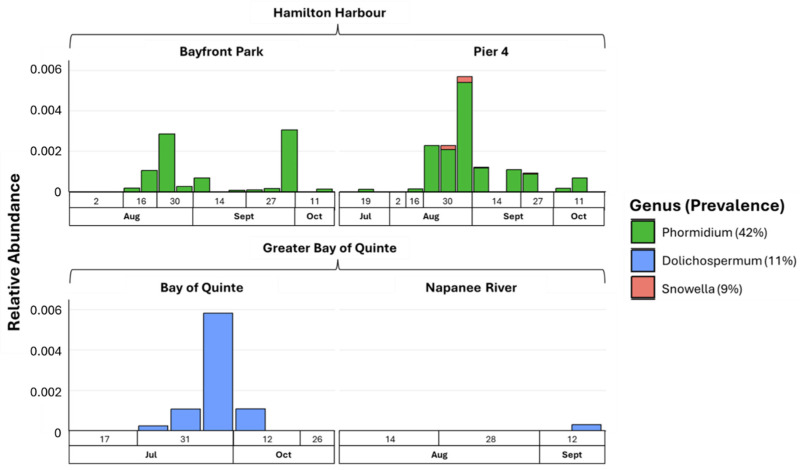
Relative abundance of microcystin-producers (except Microcystis) for all sampling sites.

To identify cyanobacterial genera associated with high levels of microcystin (*mcyE*), metabarcoding was analyzed for microcystin gene-positive samples (Figure 4). The Microcystis genus showed a 100% prevalence, accounting for 99.9% of the relative abundance. Among the lower abundant genera, *Phormidium* was dominant in the Hamilton Harbour sites, while *Dolichospermum* was associated with the Bay of Quinte sites. *Snowella* was the rarest taxon, appearing exclusively in late August and September at Pier 4. Despite the geographical proximity between the Hamilton Harbour sites, *Snowella* was not detected in Bayfront Park.

## 3. Discussion

The proliferation of cyanobacterial harmful algal blooms (cHABs) poses substantial environmental and public health risks, primarily because of the degradation of recreational water quality, leading to the release of harmful toxins into water bodies, which not only disrupt aquatic ecosystems but also endanger human health through direct contact or ingestion. Timely detection and comprehensive profiling of cyanobacteria-associated health risk factors are necessary for long-term sustainability strategies of water ecosystems. Despite the growing availability of cyanobacteria molecular technologies, cHAB detection and decision-making are commonly performed using decades-old methods, including light microscopy and quantitative enumeration of cyanobacterial cells using culturing or nanoplankton counting chambers. Although light microscopy is a cost-effective technology, it requires extensive knowledge of cyanobacterial taxonomy, suffers from limited sensitivity, and may not differentiate between cyanotoxin-producing and non-producing strains. Cyanobacterial molecular markers for water quality monitoring include direct detection/identification of cells or cyanotoxins and estimating taxonomic or cyanotoxin gene copies/transcripts [18]. However, cyanobacteria are a diverse group of organisms, and reliance on a single target assay may not reveal the temporal and longitudinal changes [19,20]. Therefore, understanding region-specific differences and underlying patterns of cHAB proliferation can allow for the development of targeted risk identification and remediation measures. This study explores the combined application of robust molecular and DNA sequencing strategies to comprehensively assess the cyanobacterial dynamics throughout the summer in association with increased microcystin concentration for different sites.

Alpha diversity matrices demonstrated little to no shift in overall bacterial diversity throughout the summer, and the values obtained were consistent with studies on North American regions, including Lake Ontario [21] and Lake Huron [22]. A study on Lake Utah [23], which is not part of the Great Lakes, observed a decrease in alpha diversity in association with bloom formation, but for our tested sites, such a change was not observed, which suggests that the underlying mechanisms of bacterial community interactions can be different from one region to another. Additionally, Three Mile Lake (Muskoka), compared with the sites (Hamilton Harbour and Bay of Quinte) in large Great Lake settings, is an inland lake that differentiated into a separate cluster, indicating a difference in the whole microbiome and cyanobiome diversity compared with the other tested sites. Differences in cyanobacterial diversity are governed by the following major environmental factors: initial phytoplankton composition, environmental variables, temperature, nutrient load, and temporal changes in the environmental variables [24,25]. Napanee River, part of the water continuum for the Bay of Quinte, showed a similar bacterial and cyanobacterial taxonomic profile to the lake system, indicating connectivity from a river to lake setting, which is similar to observations in the Lake Erie aquatic corridor [26].

Similar to a study on the Lake Erie region bacterial composition [26], our data also showed total cyanobacterial relative abundance was consistent throughout the summer, and cyanobacteria was one of the predominant bacterial groups. Despite consistent detection of cyanobacterial sequences, the rise in microcystin levels at the Hamilton Harbour sites in mid- to late summer can be due to the activation of microcystin synthesis due to environmental changes, including temperature and nutrient load [27,28]. Among the environmental changes, an increase in temperature [29], nitrogen [30], and phosphorus [31] concentrations can promote the development of cHABs. For example, an increase of 10 °C above 20 °C in temperature can promote the development of cyanobacterial biomass [32]. Microcystin concentration showed a moderate positive correlation with the cyanobacterial taxonomic marker (*16S* rRNA) for our tested sites. However, microcystin production may decrease in response to an increase in total cyanobacterial biomass [33], and the microcystin production rate can be higher for the initial bloom phase than in later stages [34]. Therefore, the relationship between total cyanobacterial biomass/concentration and cyanotoxin production can be affected by environmental/regional changes and may not be a reliable predictor of cyanotoxins in environmental waters.

Regarding cyanobacteria interactions with other bacterial groups, studies have identified an inverse relationship between cyanobacteria and actinobacteria [35,36], but we did not observe such a relationship for our Great Lakes sites. *Planktothrix*, *Microcystis*, *Cyanobium*, *Dolichospermum*, and *Lyngbya* were positively associated with microcystin production at our sites. *Lyngbya* is a benthic genus that forms a floating biomass in the water [37]. It has been previously observed in Hamilton Harbour co-dominating blooms with *Microcystis* and *Aphanizomenon* [38]. Up to 200 metric tonnes of *Lyngbya* mats have been observed to cover Great Lakes shorelines since it was first identified a decade ago [39]. Additionally, *Lyngbya* mats can retain and harbour fecal indicator bacteria [40,41], which could compromise recreational water quality, such as at our Hamilton Harbour sites. *Dolichospermum* and the saxitoxin gene were predominantly higher in Three Mile Lake (Muskoka). *Dolichospermum* can also produce saxitoxin [42,43] along with microcystin, and site-specific detection of the saxitoxin gene in the Three Mile Lake (Muskoka) samples may be related to an increase in *Dolichospermum* biomass/concentration. Cyanobacterial interactions and the underlying mechanisms of cHAB formation in water ecosystems are complex phenomena, and the molecular/taxonomic changes associated with cyanotoxin potential can be regionally or environmentally specific. Therefore, integrating newer, more robust molecular technologies can augment the water monitoring strategies for region-specific and targeted remediation measures.

## 4. Conclusions

1. Smaller inland lakes can have different environmental conditions than larger lake systems, leading to regional or environmentally specific taxonomic and molecular profiles.
2. Generic cyanobacterial toxin detection technologies may not fully assess the whole spectrum of microcystin congeners and other toxins, which may lead to an underestimation of cyanotoxin production.
3. Microcystin concentration may not be an effective predictor/indicator of other cyanotoxins, including saxitoxin, underscoring the importance of incorporating site-specific molecular testing strategies in environmental monitoring programs.
4. Rivers and associated receiving lake waters can have similar microbial profiles, indicating a continuum of cyanobacterial seeding into lake ecosystems.
5. *Dolichospermum*, *Pseudanabaena*, *Nodosilinea*, and *Cyanobium* show a regionally specific relationship with the saxitoxin gene levels, indicating their site-specific role in toxin production.
6. *Microcystis* and *Planktothrix* were consistently detected in all the tested sites among cHAB species, suggesting their dominance in bloom formation for the Great Lakes.
7. The taxonomic and functional cyanobacterial trends identified in this study can augment current recreational water monitoring programs for site/region-specific cHAB testing.

## 5. Materials and Methods

### 5.1. Study Design

**Figure 5:**
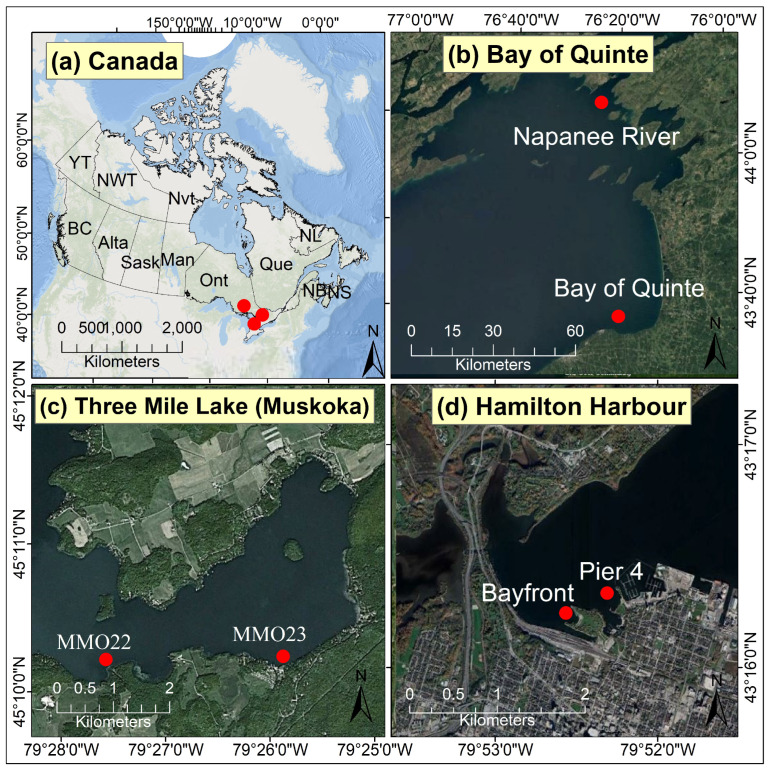
Geographical locations of the sampling sites. (**a**) Overview of the sampling locations, (**b**) Bay of Quinte, (**c**) Three Mile Lake (Muskoka), and (**d**) Hamilton Harbour.

This study focused on three specific locations in the Great Lakes region (Figure 5). The sampling locations included the Hamilton Harbour (Bayfront Park and Pier 4 Beaches), the Bay of Quinte (including the Napanee River), and the inland Three Mile Lake (Muskoka). The sampling locations were selected based on their different ecological/environmental settings compared to each other. Specifically, Hamilton Harbour sites are in close vicinity of industrial activities, Bay of Quinte sites are impacted by agricultural and urban activities, and Three Mile Lake (Muskoka) is primarily a dense cottage area.

Three biological replicates (1 m apart) were collected bi-weekly from each site in 500 mL polyethylene terephthalate (PET) bottles, and the samples were transported to the lab on ice. In total, 105 samples were collected in the summer of 2023 (July to September) and included 21 samples from each Hamilton Harbour site, 15 samples from the Bay of Quinte, 9 samples from the Napanee River, and 39 samples from Three Mile Lake (Muskoka). The samples were stored at −80 °C until processing for cyanotoxin and DNA analyses.

### 5.2. Sample Preprocessing and Nucleic Acid Extraction

The sample bottles were gently mixed to uniformly distribute the biomass before processing for cyanotoxin and nucleic acid extraction. The samples were aliquoted into 5 mL fractions into amber glass bottles for ELISA/mass spectrometry and 100 mL each for DNA and RNA extraction. The aliquoted sample was lysed by three freeze–thaw cycles at −80 °C and 35 °C to lyse the cyanobacterial cells for the USEPA 546 ELISA microcystin method [44]. Lysates were then filtered through a 0.45 µm polyethersulfone membrane filter (Cytiva, Marlborough, MA, USA), and 2–2.5 mL of filtrate was transferred to amber glass vials for the ELISA assay or long-term storage at −80 °C.

DNA and RNA extraction were performed using Norgen Plant/Fungi DNA isolation and Plant RNA extraction kits (Norgen Biotek, Thorold, ON, Canada) with minor protocol modifications. Specifically, two rounds of nucleic acid extraction were performed on each 0.45 µm filter, and two successive elution steps (20 µL each time) were performed using the same spin column filter to obtain the maximum yield of nucleic acids.

### 5.3. Enzyme-Linked Immunosorbent Assay for Microcystin Quantification

The USEPA 546 ELISA microcystin method [44] was used to quantify microcystin in the water samples. Prior to sample analysis, an Initial Demonstration of Capability (IDC) was conducted according to the USEPA Method 546 [44]. Microcystin-LR (MC-LR) standards (10 µg/mL in methanol) were obtained from Gold Standard Diagnostics (Davis, CA, USA). The MC-LR standard was diluted to a concentration of 100 ng/mL and stored at −20 °C for use in spiking the Laboratory Fortified Blank (LFB) to measure internal recovery, Laboratory Fortified Sample Matrix (LFSM), and Laboratory Fortified Sample Matrix Duplicate (LFSMD). Seven Laboratory Fortified Blanks (LFBs) were spiked with 0.5 µg/mL of MC-LR to evaluate precision and accuracy, then lysed, filtered, and assayed. Five Laboratory Reagent Blanks (LRBs) were processed similarly in the same batch as the LFBs, including a Low-Range Calibration Verification (Low-CV) control to ensure an acceptable system background. To verify the minimum reporting limit (MRL), seven LFBs were fortified at the proposed MRL concentration (0.40 ng/mL), lysed, filtered, and assayed in an analysis batch that included one low-CV, one quality control sample (QCS), and two LRBs. The analysis batch included the following quality control elements: one quality control sample (QCS, 0.75 ng/mL), one Laboratory Reagent Blank (LRB), two LFBs, one LFSM, and one LFSMD. A calibration curve (0, 0.15, 0.40, 1.0, 2.0, and 5.0 ng/mL) was generated alongside each week’s samples and was used to calculate the MC/NOD concentration in the sample duplicates. The average concentration of the two duplicates was then calculated and recorded as the final concentration of MC/NOD for each sample.

### 5.4. Analyses of Cyanotoxins and Other Bioactive Secondary Metabolites

Cyanobacterial bioactive metabolites, including cyanotoxins, were measured using mass spectroscopy methods adapted from a previous study on mass spectrometry of cyanobacterial metabolites [45]. The method reporting limits (MRLs) for anatoxins and cylindrospermopsins were between 20 and 500 ng/L (ppt), and for the microcystins and non-microcystin cyanopeptides, they were 5 or 10 ng/L (ppt). Mass spectroscopy was also used to detect and quantify saxitoxins (including gonyautoxins), and methods were adapted from a recent study [46]. The detection limit for neosaxitoxin, gonyautoxin 1&4, and gonyautoxin 2&3 was 1 ppb, 0.5 ppb, and 0.5 ppb, respectively.

### 5.5. Quantitative/Real-Time PCR for Cyanotoxins

The concentration of cyanobacterial and cyanotoxin (microcystin, saxitoxin, and cylindrospermopsin) gene copies was determined using the CyanoDTec Total Cyanobacteria and Toxin Kit (Phytoxigene^TM^, Akron, OH, USA) following the manufacturer’s instructions. A total of 50 DNA samples corresponding to samples with detectable microcystin concentration were analyzed for cyanobacteria/toxin gene copies. Four standard curves were individually run for each assay (total cyanobacteria and each toxin), and then a composite standard curve was prepared. The standard curve range for each assay spanned from 10 to 100,000 gene copies. Each reaction included 20 µL of mastermix/primer–probe solution and 5 µL of DNA from the pooled samples, the same DNA used for shotgun sequencing. Gene copy numbers for each sample were calculated using the slope–intercept equation from the standard curve and normalized to gene copies per nanogram of DNA. A gene was considered present in a sample if the gene copies/threshold-cycle values fell within the range of the standard curve, and results were only accepted if the internal amplification control threshold-cycle (Ct) value for a sample did not differ by more than 1.5 compared to the non-template control. Primer sequences from a previous study [47] were used to calculate the number of microcystin transcripts in the samples. SuperScript IV Reverse Transcriptase (Thermo Scientific, Waltham, MA, USA) was used for first strand synthesis, followed by qPCR using SsoAdvanced Universal SYBR Green Supermix (BioRad, Hercules, CA, USA), and each reaction constituted 5.0 µL of cDNA, 2.0 µL of both forward and reverse primers (10 nM), 12.5 µL of master mix, and 3.5 µL of nuclease-free water. The standard curve (range: 10 to 40,000 copies) was used to calculate the number of transcripts in the reaction. Microcystin transcripts were normalized per microlitre of the sample using the RNA quantification and the cDNA generation efficiency of SuperScript IV Reverse Transcriptase [48], followed by the normalization of gene transcripts per nanogram of RNA used in each reaction.

### 5.6. Primer Design and Metabarcoding Sequencing Library Preparation

Microcystin (*mcyE*) metabarcoding was performed to identify microcystin-producing cyanobacterial genera in the samples, while *16S* metabarcoding was used to characterize the bacterial taxonomic profile. For microcystin metabarcoding, a primer set targeting *mcyE* hypervariable regions from a previous study [49] was used with minor modifications to identify potential microcystin-producing cyanobacterial taxa. Specifically, four degenerate bases were added to the primers after alignment against the NCBI non-redundant database (Accessed 30 October 2023) to capture a broad range of microcystin-producing cyanobacterial taxa. The modified primer sequences were as follows: Forward Primer = 5’-TT TGG RGT TAA CTT TTT TGG BCA TAG TC-3’, and Reverse Primer = 5’-TAA TTC TTK AGG YTG TAA ATC KGG TTT-3’. For *16S* metabarcoding, the library preparation guide from Illumina accessible at https://support.illumina.com/documents/documentation/chemistry_documentation/16s/16smetagenomic-library-prep-guide-15044223-b.pdf (accessed on 1 January 2023) was used. The purified DNA from each sample was normalized to 5.0 ng/µL and used for amplicon PCR to avoid concentration bias effects. The amplicon PCR reaction mix included 2.5 µL of genomic DNA, 5.0 µL of each primer (10 nM), and 12.5 µL of DreamTaq™ Hot Start Green PCR Master Mix (Thermo Fisher Scientific, Waltham, MA, USA). The amplicon PCR was executed in a thermal cycler with the following program: initial denaturation at 95 °C for 3 min, followed by 25 cycles of 95 °C for 30 s, 55 °C for 30 s, and 72 °C for 30 s, with a final extension at 72 °C for 5 min. PCR products were purified using 20.0 µL of Ampure XP magnetic beads (Beckman Coulter, Brea, CA, USA).

The index PCR reaction mix consisted of 2.5 µL of purified amplicon DNA, 8.0 µL of nuclease-free water, 12.5 µL of DreamTaq™ Hot Start Green PCR Master Mix (Thermo Fisher Scientific, Waltham, MA, USA), and 2.0 µL of NEBNext^®^ Multiplex Oligos 96 Unique Dual Index Primer Pairs for Illumina^®^ (New England Biolabs, Ipswich, MA, USA). Index PCR was conducted in a thermal cycler (BioRad CFX96) with the following program: initial denaturation at 95 °C for 3 min, followed by 8 cycles of 95 °C for 30 s, 55 °C for 30 s, and 72 °C for 30 s, with a final extension at 72 °C for 5 min, followed by final purification using Ampure XP beads (Beckman Coulter, Brea, CA, USA). DNA libraries were sequenced on the Illumina NextSeq 1000 platform at the Farncombe Metagenomics Facility at McMaster University (Hamilton, ON, Canada).

### 5.7. Data Analysis and Bioinformatics

Approximately 300,000 DNA sequences were obtained for each sample for both *16S* and *mcyE* metabarcoding. Sequence quality was assessed using FastQC [50]. Primer trimming, quality filtration, dereplication, denoising, chimera removal, and merging were conducted with DADA2 [51] in QIIME2 [52] to produce amplicon sequence variants (ASVs). To ensure the quality of novel *mcyE* amplicons, the resulting ASVs were translated and aligned using MUSCLE [53], followed by the manual removal of partial genes and spurious amplicons.

The *16S* rRNA ASVs were referenced using the full-length Greengenes2 database [54]. A custom database for all microcystin synthetase (*mcy*) cluster genes was compiled using sequences from NCBI Nucleotide (accessed on 27 March 2024). The built-in Naïve-Bayes q2-feature-classifier in QIIME2 [27] was trained on these databases with the respective gene primers and assigned taxonomy to the ASVs. The resulting feature tables were exported into a phyloseq [55] object in R for data manipulation and taxonomic analysis. Core phyla of the bacterial microbiome were identified using a threshold of 80% prevalence and at least 1% relative abundance [56]. Here, we use the term “Cyanobiome”, to describe the core cyanobacterial genera identified with a threshold of 50% prevalence and 1.0% relative abundance in all the samples from a particular location [57].

For downstream analysis, ASV counts were normalized using Cumulative Sum Scaling in the metagenomeSeq package [58]. Diversity analysis was conducted using the vegan package [34], correlation analysis using the microbiome package [59], and differential abundance analysis using the metagenomeSeq package [60]. Shapiro–Wilk’s normality test was applied to all the taxonomic and molecular markers information prior to Pearson rank correlation analysis [61]. Differential abundance was calculated using a zero-inflated log-normal mixture model [62], with Benjamini–Hochberg adjustment for a false discovery rate correction [63].

## Figures and Tables

**Table 1:** Detection frequency of microcystin congeners and other cyanotoxins at Hamilton Harbour (*n* = 19), Bay of Quinte (*n* = 10), and Three Mile Lake (*n* = 17).

Microcystin Congener/ Cyanotoxin	Hamilton Harbour (Detection Frequency)	Bay of Quinte (Detection Frequency)	Three Mile Lake (Detection Frequency)
MC-RR	95%	30%	ND
MC-YR	79%	ND	ND
MC-HtyR	ND	ND	ND
MC-LR	89%	30%	ND
MC-HilR	ND	ND	ND
MC-WR	ND	ND	ND
MC-LA	5%	10%	ND
MC-LY	ND	ND	ND
MC-LW	ND	ND	ND
MC-LF	ND	ND	ND
Anabaenopeptin B	26%	ND	6%
Anabaenopeptin A	37%	ND	ND
Oscillamide Y	37%	ND	ND
Anatoxin A	63%	ND	ND
Cylindrospermopsin	ND	ND	ND

**Table 2:** Correlation between cyanobacteria and cyanotoxin indicators. Gene copies and transcripts were calculated as copies/mL of samples.

Cyanobacteria/Cyanotoxin Metrics		Correlation Coefficient	*p*-Value
Microcystin	*mcyE* Gene Copies	0.71	7.9^−7^
	Cyanobacteria *16S*	0.50	1.4^−3^
	*sxtA* Gene Copies	0.23	0.34
	*mcyE* Transcripts	0.52	4.4^−3^
*mcyE* Transcripts	*mcyE* Gene Copies	0.62	5.8^−4^
	Cyanobacteria *16S*	0.31	0.08
	*sxtA* Gene Copies	0.05	0.72

## Data Availability

The data presented in this study are available upon request from the corresponding author because of sponsor privacy restrictions.

## Supplementary Materials

The following supporting information can be downloaded at https://www.mdpi.com/article/10.3390/toxins16110471/s1: Figure S1. Comparison of microcystin gene copies among the sampling locations; Figure S2. Comparison of microcystin transcripts among the sampling locations; Figure S3. Comparison of saxitoxin gene copies among the sampling locations; Figure S4. Comparison of cyanobacterial 16S gene copies among the sampling locations; Figure S5. Venn diagram analysis of shared and unique amplicon sequence variants (ASVs) between the sampling locations; Figure S6. Relative abundance comparison of bacterial phyla for all sampling locations throughout the summer; Figure S7. Relative abundance comparison of cyanobacterial genera for all sampling locations throughout the summer; Figure S8. Correlation matrix analysis between cyanobacterial molecular markers and identified cyanobacterial genera; Table S1. Alpha diversity analysis for tested sites/locations using whole microbiome information; Table S2. Alpha diversity analysis for tested sites/locations using Cyanobiome information.
