# Supplementary material for: Regional and Longitudinal Dynamics of Cyanobacterial Blooms/Cyanobiome and Cyanotoxin Production in the Great Lakes Area
Source: Toxins (Basel). 2024 Nov 1;16(11):471. doi: 10.3390/toxins16110471 (PMC11598720; doi:10.3390/toxins16110471)
Supplement: Supplementary file 1 [file toxins-16-00471-s001.zip › toxins-3260650-supplementary.pdf]

toxins

MDPI

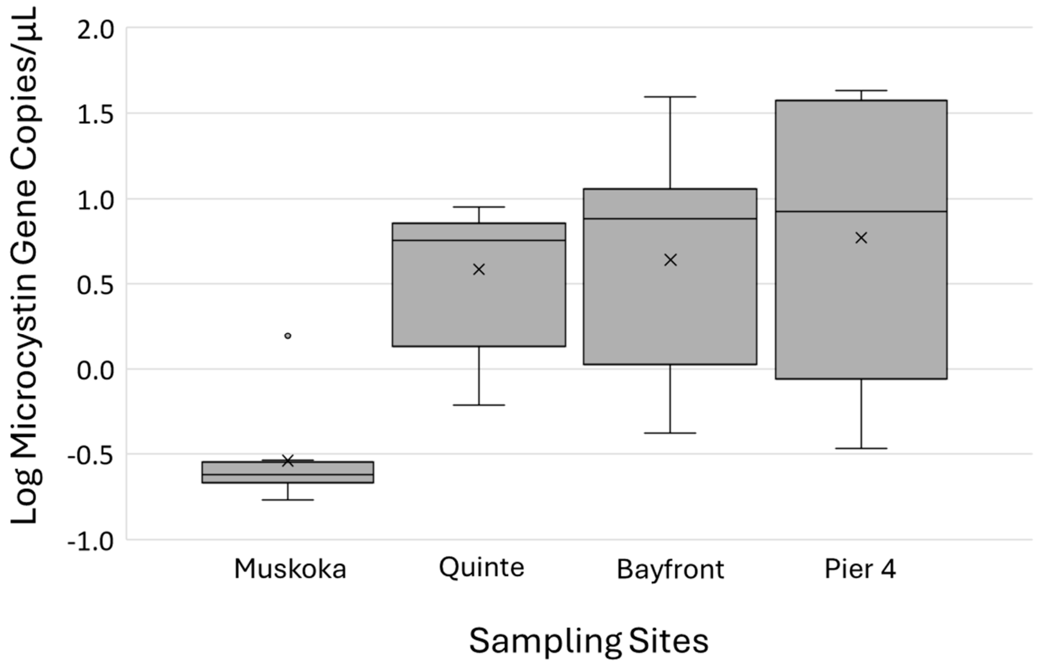

Figure S1. Comparison of microcystin gene copies among the sampling locations. Log gene copies are calculated as per microlitre of water samples. Sampling sizes for each sampling location included Bayfront Park (n = 8), Pier 4 (n = 8), Bay of Quinte (n = 8), and Three Mile Lake (Muskoka) (n = 13).

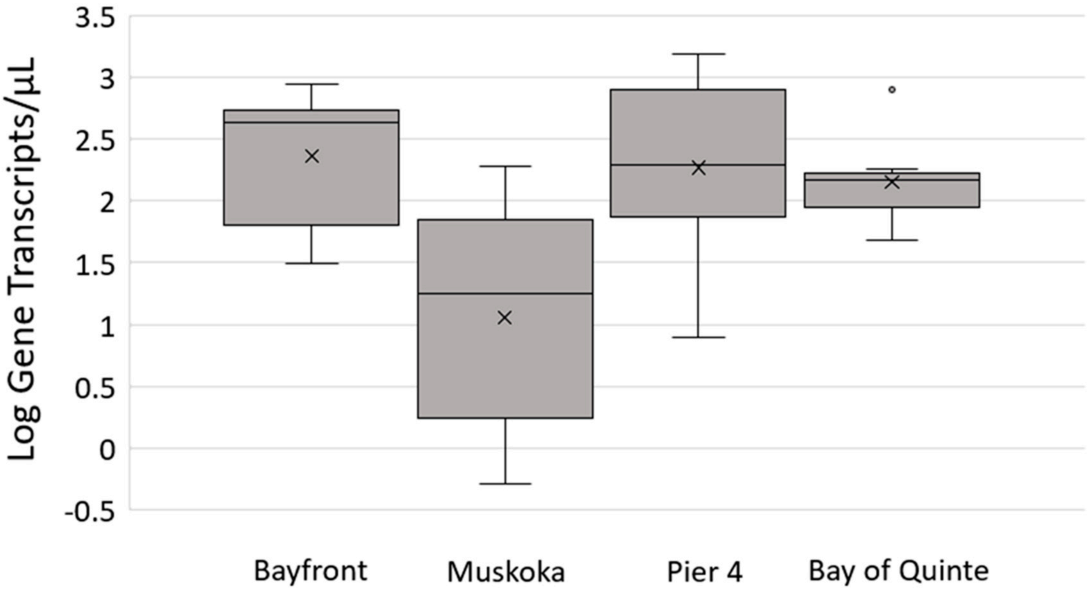

Figure S2. Comparison of microcystin transcripts among the sampling locations. Log gene transcripts are calculated as per microlitre of water samples. Sampling sizes for each sampling location included Bayfront Park (n = 10), Pier 4 (n = 11), Bay of Quinte (n = 10), and Three Mile Lake (Muskoka) (n = 10).

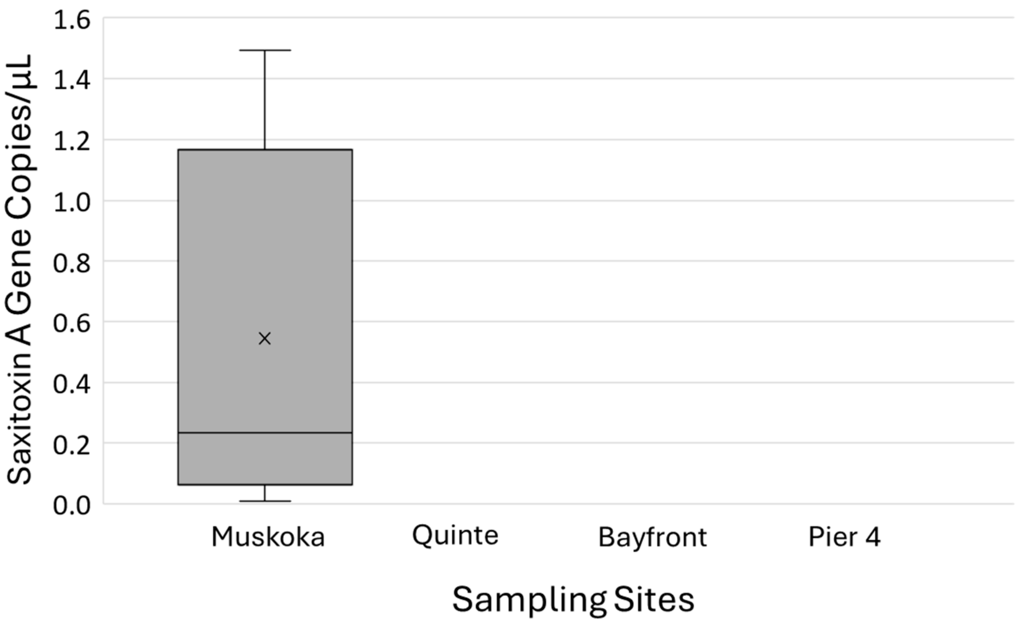

Figure S3. Comparison of saxitoxin gene copies among the sampling locations. Log gene copies are calculated as per microlitre of water samples. Sampling sizes for each sampling location included Bayfront Park (n = 8), Pier 4 (n = 8), Bay of Quinte (n = 8), and Three Mile Lake (Muskoka) (n = 13).

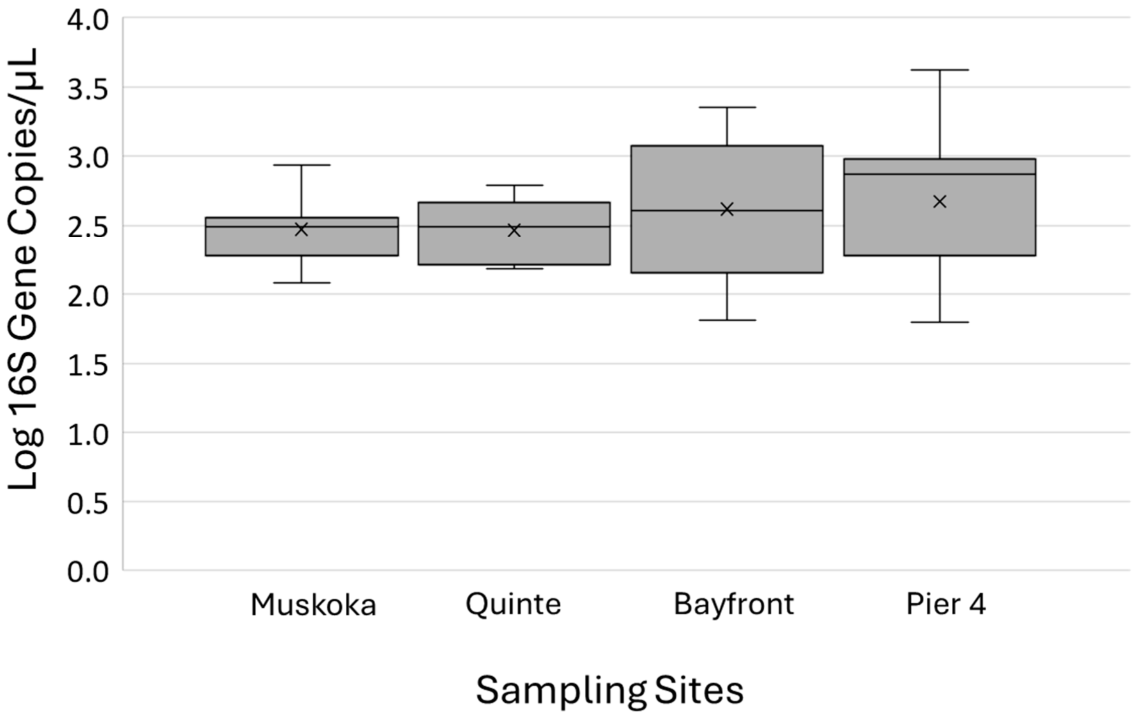

Figure S4. Comparison of cyanobacterial 16S gene copies among the sampling locations. Log gene copies are calculated as per microlitre of water samples. Sampling sizes for each sampling location included Bayfront Park (n = 8), Pier 4 (n = 8), Bay of Quinte (n = 8), and Three Mile Lake (Muskoka) (n = 13).

Table S1. Alpha diversity analysis for tested sites/locations using whole microbiome information.

Sampling Sites	No. of Taxa Identified	Shannon-Weaver Diversity Matrix	Simpson-reciprocal Diversity Matrix
**Bayfront Park (n = 21)**	1390 ± 375	5.0 ± 0.36	0.97 ± 0.007
**Pier 4 (n = 19)**	1534 ± 465	5.1 ± 0.26	0.97 ± 0.006
**Bay of Quinte (n = 13)**	1228 ± 250	5.0 ± 0.26	0.97 ± 0.016
**Napanee River (n = 8)**	1527 ± 299	5.2 ± 0.36	0.96 ± 0.051
**Three Mile Lake (n = 33)**	1516 ± 663	5.2 ± 0.44	0.98 ± 0.008

Table S2. Alpha diversity analysis for tested sites/locations using Cyanobiome information.

Sampling Site	No. of Taxa Identified	Shannon-Weaver Diversity Matrix	Simpson-reciprocal Diversity Matrix
**Bayfront Park (n = 21)**	104 ± 36	2.5 ± 0.72	0.78 ± 0.15
**Pier 4 (n = 19)**	127 ± 34	2.8 ± 0.62	0.82 ± 0.11
**Bay of Quinte (n = 13)**	163 ± 36	3.3 ± 0.95	0.82 ± 0.20
**Napanee River (n = 8)**	208 ± 21	3.2 ± 0.87	0.83 ± 0.19
**Three Mile Lake (n = 33)**	164 ± 54	3.2 ± 0.49	0.87 ± 0.07

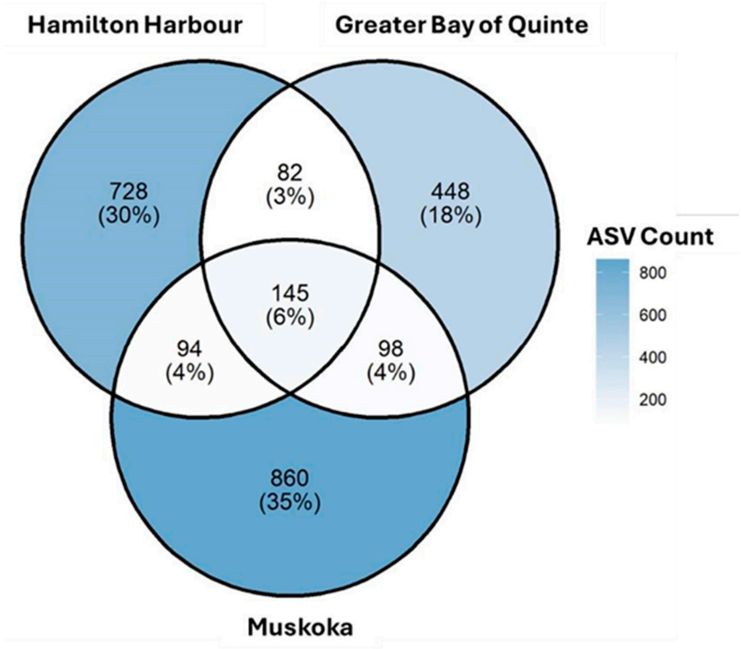

Figure S5. Venn diagram analysis of shared and unique amplicon sequence variants (ASVs) between the sampling locations.

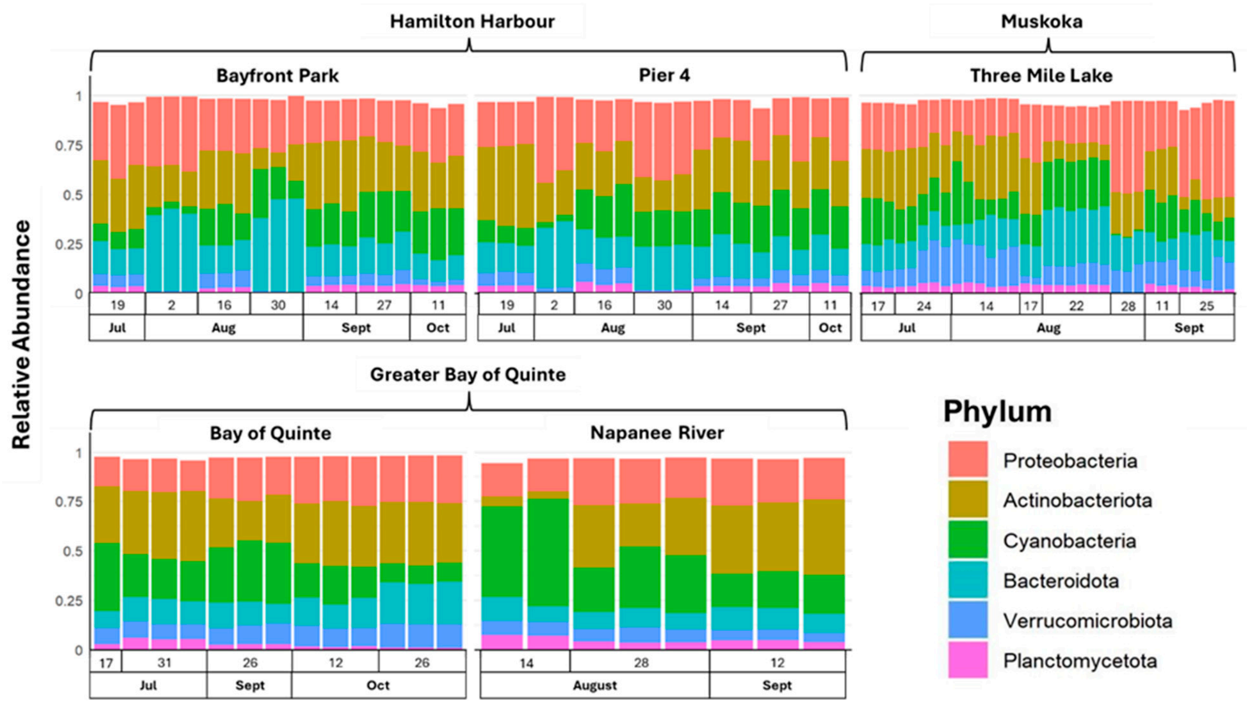

Figure S6. Relative abundance comparison of bacterial phyla for all sampling locations throughout the summer.

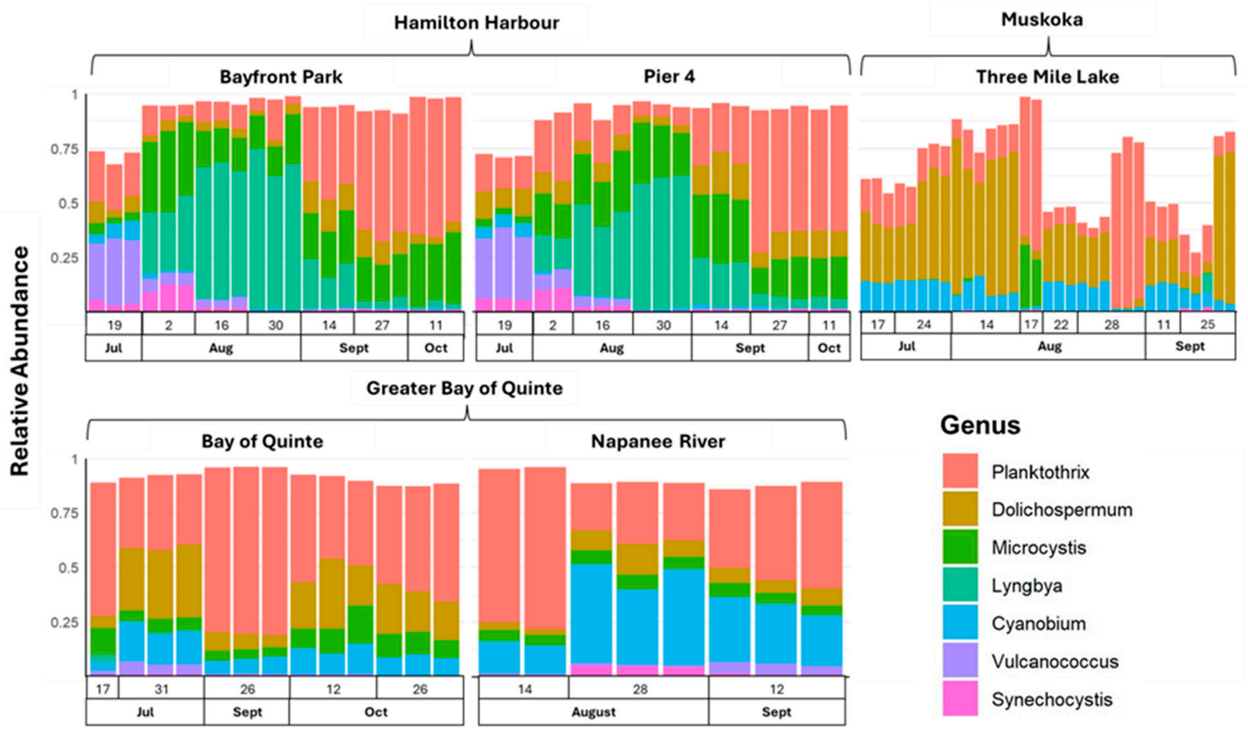

Figure S7. Relative abundance comparison of cyanobacterial genera for all sampling locations throughout the summer.

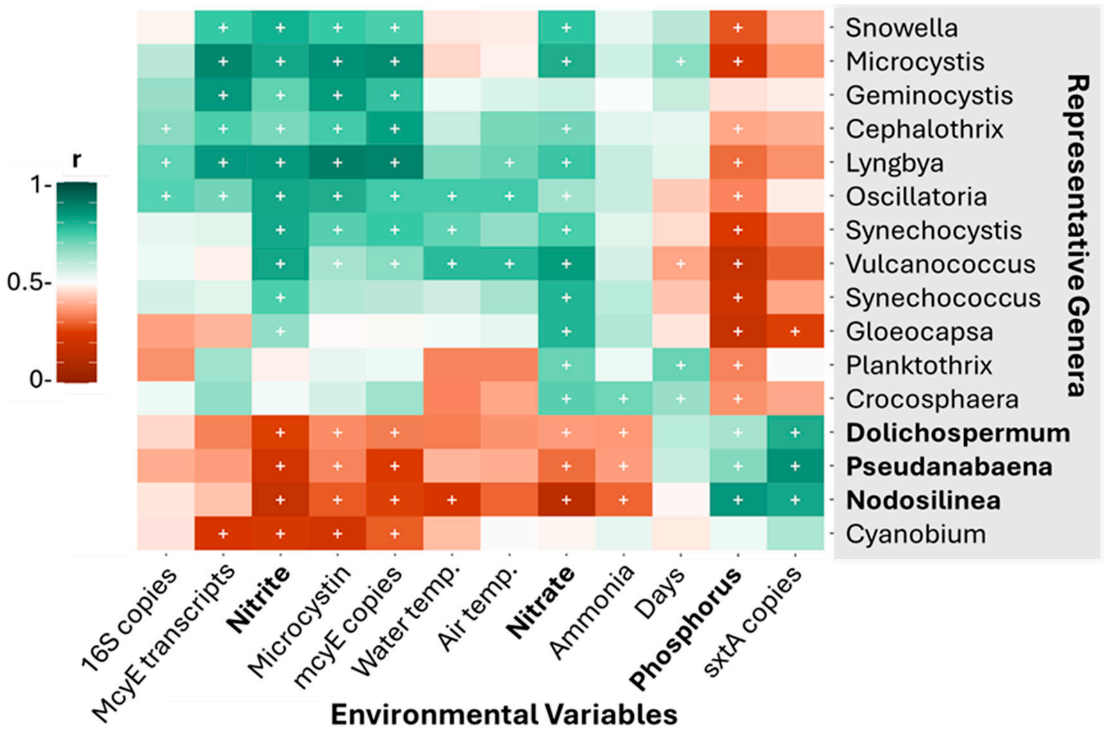

Figure S8. Correlation matrix analysis between cyanobacterial molecular markers and identified cyanobacterial genera.
